# Efficient dye removal from industrial wastewater using sustainable activated carbon and its polyamide nanocomposite derived from agricultural and industrial wastes in column systems

**DOI:** 10.1039/d3ra03105e

**Published:** 2023-08-22

**Authors:** Ahmed M. Zayed, Bahaa S. Metwally, Mostafa A. Masoud, Mahmoud F. Mubarak, Hussain Shendy, Mahmoud M. Abdelsatar, Petros Petrounias, Ahmed H. Ragab, Abeer A. Hassan, Mahmoud S. M. Abdel Wahed

**Affiliations:** a Applied Mineralogy and Water Research Lab (AMWRL), Geology Department, Faculty of Science, Beni-Suef University Beni Suef 62521 Egypt ahmed.zayed@science.bsu.edu.eg mostafa_masoud@science.bsu.edu.eg; b Textile Technology Department, Faculty of Technology and Education, Beni-Suef University Beni-Suef 62521 Egypt; c Petroleum Application Department, Egyptian Petroleum Research Institute 1 Ahmed El-Zomor Street, El-Zohour Region, Nasr City Cairo 11765 Egypt; d Chemical Process & Energy Resources Institute, Centre for Research & Technology Hellas (CERTH) 15125 Athens Greece; e Chemistry Department, College of Science, King Khalid University P.O. Box 9004 Abha 61413 Saudi Arabia

## Abstract

Sugar beet crown (SBC) waste was employed to produce sustainable activated carbon (AC) by a thermo-chemical activation procedure using a fixed ratio of H_3_PO_4_/SBC (1 : 1 w/w ratio) at 550 °C/2 h. An activated carbon/polyamide nano-composite (AC/PA) was also prepared through the polymerization of the fabricated AC (90%) with polyamide (PA, 10%) synthetic textile waste using a proper dissolving agent at a specified w/w ratio with the employed polymer (formic acid/PA = 82/18%). Both AC and its derivative AC/PA were employed in the remediation of dyes from industrial wastewater in column systems, and their efficiencies were compared at various applied experimental conditions. The adsorption of the industrial dye waste (IDW) was a pH-, flow rate-, and bed thickness-controlled process by the regarded adsorbents. Kinetic studies confirmed the suitability of the Thomas equation over the Yoon and Nelson model in predicting the dynamic adsorption process of IDW by AC and AC/PA as was assured by the close agreement among the calculated and experimental uptake capacities of both adsorbents at the same applied flow rates, suggesting the chemisorption nature of IDW adsorption. Additionally, electrostatic attraction was the leading mechanism of IDW adsorption by AC and AC/PA composite with some advantages of the former over the latter.

## Abstract 2

The fabrication process of AC from SBC (a) and AC/PA composite from AC and PA (b). The treatment process of the IDW using both AC and the AC/PA composite (c).

## Graphical abstract

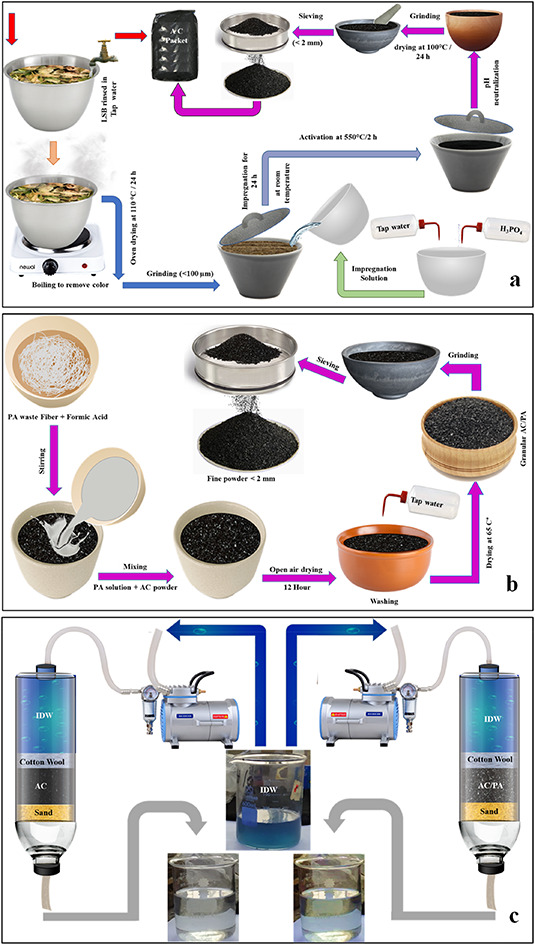

## 1. Introduction

According to their origin, structure, and uses, dyes are categorized into several types such as azo, direct, reactive, mordant, acid, basic, disperse, and sulfide dyes^1,2^ and are commonly used in textile industries. Additionally, these dyes are applied in a variety of industries, such as the production of batteries, food, paper, and leather tanning, producing large amounts of colored wastewater.^3^ These wastes are hazardous to the environment, living species, and human health due to their high concentrations of organic pollutants that are extremely toxic, and they have disagreeable taste, odor, and color. About 7 × 10^7^ tons of synthetic dyes are produced worldwide annually, with over 10 000 tons of such dyes used by textile industries.^4^ The untreated release of dyes into aquatic ecosystems, such as lakes, rivers, and streams, poses major ecotoxicological risks and has a toxic effect on living things.^5^ Therefore, the world will need to solve this industrial wastewater problem with different methods.

Precipitation, coagulation, filtration, ion exchange, advanced oxidation, bioremediation, and activated sludge mechanisms represent some of the physical, chemical, and biological technologies used for dye removal from wastewater.^6–9^ Comparing adsorption with other techniques, it is important to consider their specific strengths and limitations. For instance, chemical oxidation can be highly effective in removing certain organic pollutants using strong oxidizing agents. Bioremediation, on the other hand, utilizes microorganisms to degrade organic pollutants, making it suitable for certain types of wastewaters.^8,10^ Activated sludge mechanisms involve the use of biological processes for the treatment of wastewater, and it is particularly effective in removing organic matter. However, most of these traditional treatment procedures are undesirable because of their operational complicity and costs,^11^ causing system corrosion, hazardous by-product production, consumption of chemicals, pH sensitivity, sludge generation, as well as disintegration of the microorganisms and organic materials.^12–14^ Additionally, some of these procedures (biological ones) may raise the concentrations of biochemical oxygen demand (BOD) and chemical oxygen demand (COD) in the treated water. Regarding filtration, the filter openings are prone to clogging.^15^ Conversely, the adsorption process is the most recommended treatment strategy due to its high efficiency, applicability in a wide pH range, low cost, simplicity, and viability for large-scale applications.^10,11,16^

To carry out adsorption studies and to determine the essential parameters for controlling the removal of contaminants from wastewater, both batch and column methods are necessary.^17^ Fixed bed adsorption column represents one of the most popular and effective methods for removing contaminants from water. However, batch adsorption is preferred in laboratory-scale experiments as it requires less time, only a small amount of material, and is used to calculate the maximum adsorption capacity of the adsorbent.^18^ The column system is preferable to batch adsorption because of its simple operation, high contact time with the adsorbent, quicker adsorption process, and higher amount of adsorbate, so it can be applied at a large scale rather than a batch system.

Activated carbon (AC), also known as activated charcoal, is carbon that has been processed to have small, low-volume pores that increase the surface area available for adsorption or chemical reactions. One of the two following methods can be used to activate carbon: (1) physical activation, in which the raw material is transformed into activated carbon using hot gases. Air is then introduced to burn out the gases, creating a graded, screened, and de-dusted form of activated carbon. Generally, one or more of the following procedures are used to accomplish this: (a) biomass carbonization, which involves pyrolyzing the carbon-containing material at temperatures between 600 and 900 °C,^19^ often in an inert environment with gases such as argon or nitrogen. (b) Activation/oxidation, which is the second stage, aims to provide high internal porosity and surface area to enable and enhance the adsorption function. At temperatures above 250 °C, usually in the range of 600–1200 °C, raw material or carbonized material is exposed to oxidizing atmospheres (oxygen or steam). The activation is performed by heating the sample for 1 h in a muffle furnace at 450 °C in the presence of air.^20^ (2) Chemical activation, in which the carbon material is impregnated with certain chemicals. According to,^21^ the chemical is often an acid, a strong base, or a salt, such as phosphoric acid, potassium hydroxide, sodium hydroxide, potassium carbonate, calcium chloride, and zinc chloride. Then, the carbon is heated to high temperatures (between 250 and 600 °C). The temperature is believed to activate the carbon at this stage by forcing the material to open up and have more microscopic pores. Due to lower temperatures, higher quality consistency, and quicker activation times, chemical activation is preferred to physical activation.^22^

AC is produced from carbonaceous source materials such as bamboo, coconut husk, willow peat, wood, coir, lignite, coal, and petroleum pitch. Owing to their properties, it is also utilized in a variety of applications, including medicine, metals recovery, the food and beverage industry, acute intoxication treatment, water purification, biogas purification, and air emission purification. In water treatment, AC adsorption is now thought to be the most common and efficient physical approach,^23–25^ as it is composed of a microporous, homogenous structure with a high surface area and radiation stability.

Recently, there has been a global trend towards the valorization of unexploited materials and waste as a means to address the issue of environmental pollution.^7,22,26–32^ One such approach is the valorization of some agricultural wastes such as sugar beet to produce AC.^33,34^ Sugar beet is one of the most important sugar-producing plants worldwide, as the root contains a lot of sucrose. About 11 million tons of sugar beets were produced in Egypt in 2018.^35^ The root contains about 75% water, 20% soluble solids, 16% sucrose, and 4% nitrogenous and mineral salts. These salts, particularly sodium and potassium salts, prevent sugar from crystallizing and reduce crop quality.^35^ The leaves are numerous and broad, growing in a tuft from the crown of the beet, which is usually level with or just above the ground surface. SBC (sugar beet crown that includes stem & leaves) is mainly composed of cellulose, hemicellulose, pectin, and lignin, which reflects the enrichment with carbohydrates, fibers, proteins, and elements, such as sodium, potassium, calcium and iron.^36^ Additionally, SBC has a high ratio of carbon that makes it available as a raw material for activated carbon synthesis.^37^ Moreover, SBC is a cost-effective raw material compared to some other carbon sources. The annual landfill dumping of SBC in huge volumes may contribute to some environmental issues since the SBC decomposition emits unpleasant odors and attracts pests, posing health risks. Additionally, the waste produces greenhouse gases like methane, contributing to climate change if not managed effectively.

Polyamide (PA), commonly known as nylon, occurs both naturally and artificially. Polyamide fiber waste is a cheap product resulting from textile factories that are characterized by high durability and strength. The incorporation of PA in the activated carbon composites can enhance their durability and strength. Additionally, the availability of PA wastes for low costs can reduce the overall production expenses of the activated carbon composites. However, the improper disposal of PA wastes as synthetic fibers can cause environmental pollution, as these wastes are not easily biodegradable and can accumulate in ecosystems. PA wastes can also break down into microplastics, contaminating soil, water, and air, posing risks to the environment.^38^ Furthermore, PA wastes may contain chemical residues that can leach into the environment, causing toxic effects on organisms and disrupting hormonal systems.

Therefore, the current work aims to: (1) valorize the negative environmental impact of SBC as an agro-residue through its conversion into valuable AC *via* thermo-chemical activation process at 550 °C/2 h; (2) reduce the deleterious effect of PA through its incorporation in the production of the AC/PA nanocomposite *via* polymerization process; (3) compare the efficiency of both derivatives (AC & AC/PA) in the remediation of dyes from industrial wastewater using a column system under different experimental parameters (pH, bed thickness, flow rate).

## 2. Materials and methods

### 2.1. Raw materials

**Fig. 1:**
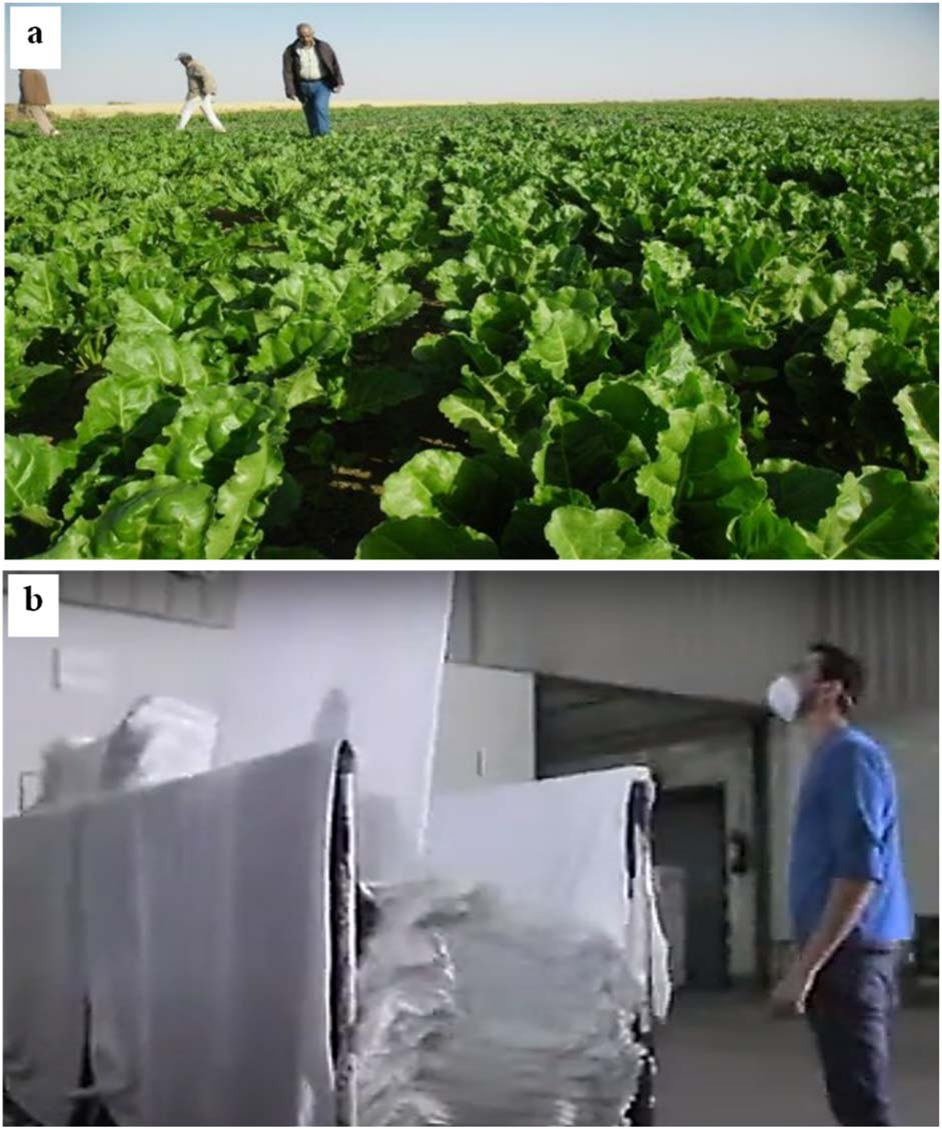
Sugar beet crown (SBC) collected from farms cultivating the sugar beet crop at Beni-Suef Governorate (a); polyamide (nylon) waste collected from the Salamtext Factory (b).

Sugar beet crown waste (SBC) samples were compiled from cultivated areas with sugar beet crops in the Deshasha area, Beni-Suef Governorate during the harvesting period (Fig. 1a). These SBC samples were required to produce valuable activated carbon. Polyamide 6 (PA6) samples, which are considered one of the most common polymer plastics and/or man-made yarn industrial wastes, were collected from Salamtext Factory, Industrial Zone of El Obour City, Egypt (Fig. 1b). The PA6 samples are important for the conversion of the prepared AC into the AC/PA composite.

### 2.2. Preparation of the collected raw materials

The SBC samples were washed with tap water (TW) to remove any adhered deleterious materials such as dust. The washed sample was boiled until the colored components were removed. After oven drying at 105 °C/24 h, the SBC samples were ground to obtain the required sizes (<100 μm).

The PA fiber waste was washed with a 1 g l^−1^ nonionic detergent solution at 80 °C for 20 min, and rinsed thoroughly with distilled water to remove any surface impurities before air drying.^39^

### 2.3. Synthesis of active carbon from SBC

**Fig. 2:**
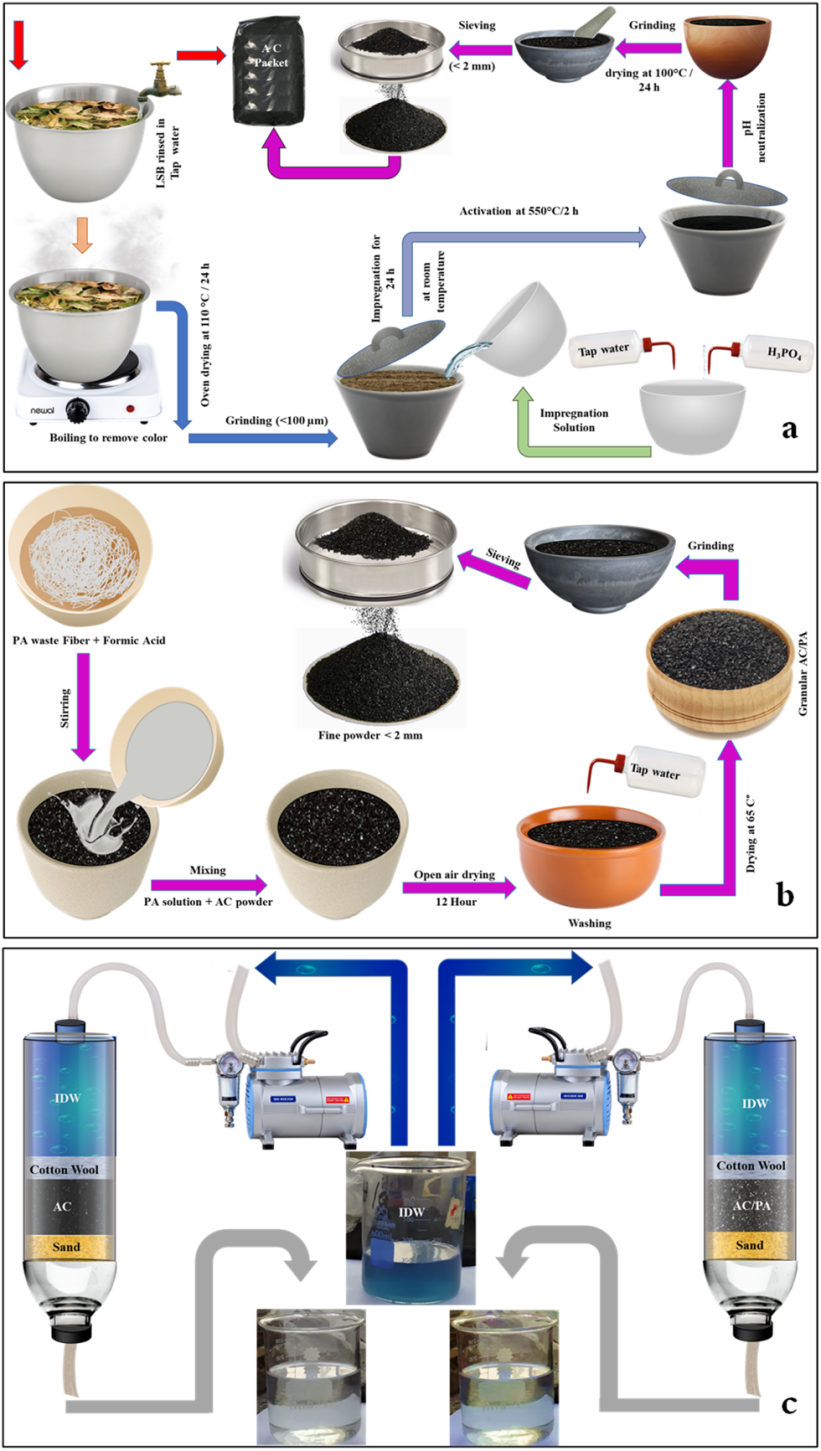
Illustration of the fabrication process of AC from SBC (a), and of the AC/PA composite from AC and PA (b). The treatment process of the IDW using both AC and AC/PA composite (c).

For AC synthesis, 50 g of the prepared SBC powder was mixed with an equal weight (50 g) of H_3_PO_4_ (85%, E. Merck, Germany), to achieve a 1 : 1 (H_3_PO_4_/LSB) w/w solid-to-liquid ratio. Then, 200 ml of TW was employed to liquefy the prepared blend, which was left uninterrupted at ambient temperature overnight. The permeated sample was poured into a refractory crucible to be activated at 550 °C/2 h in a programmable muffle furnace with a heating rate of 10 °C min^−1^.^40^ The produced AC samples were thoroughly washed with 0.1 M NaOH, and then by TW numerous times to remove any trapped H_3_PO_4_ residue until the supernatant pH become neutral. After oven drying at 100 °C overnight, the AC sample was ground in mortar and pestle to <3 mm and then tightly packed for the following deployment (Fig. 2).

### 2.4. Fabrication of the AC/PA composite

For the preparation of AC/PA, the prepared PA6-fiber waste was dissolved in formic acid (90%), with a fixed concentration (18% w/w), according to the protocol followed by.^39^ So, 1.8 g of PA was dissolved in 8.2 g of formic acid to maintain a previously reported ratio of 18%. This mixture was homogenized *via* a stirring process at ambient temperature until a homogeneous/viscous solution was obtained. Then, 16.2 g of the prepared AC was added to the previous mixture with continuous stirring also at room temperature, achieving a w/w impregnation ratio of 10 : 90% (PA6 : AC). This PA6 ratio (10%) was selected to fulfill the minimal reduction ratio in the geometric parameters, especially surface area, and inherited porous structure. Such mixture was left in open air overnight to permit evaporation of the excess formic acid. After drying, the produced composite was washed thoroughly several times with TW until pH neutralization was achieved, and the product was oven-dried at 65 °C for 12 h. The produced AC/PA composite was re-ground using mortar and pestle to obtain fine grains (<3 mm). The collected powder was packed in an airtight container for further application. The fabrication process of the AC/PA composite is summarized in Fig. 2b.

### 2.5. Characterization of SBC, AC, and the AC/PA composite

The agricultural waste (SBC) and derivative products (AC & AC/PA composite) were characterized by XRD (APD-3720/Philips diffractometer with Cu Kα radiation/operated at 40 kV and 20 mA/scanning speed 5° per min/range of 2*θ* from 5° to 80°), scanning electron microscopy (SEM, JEOL/JSM-6700F/Japan/20–30 kV beam energy/11.10–12.20 mm as a working distance) supplied with energy-dispersive X-ray spectrometry (EDS), and FT-IR (FTIR-2000/Bruker spectrometer/reflection mode at 4 cm^−1^ resolution/scanning range from 400 to 4000 cm^−1^) techniques to determine their crystalline phases, morphological features, elemental composition, and the distinctive functional groups, respectively. The *S*_BET_ (BET surface area), *V*_t_ (total pore volume), and *D*_p_ (average pore diameter) of SBC, AC and the AC/PA composite were determined after vacuum degassing at 100 °C for 2 h *via* Quantachrome Surface Area Analyzer (Nova 2000). The BJH (Barrett–Joyner–Halenda) equation^41^ was employed to estimate *D*_p_ and *V*_t_, whereas *S*_BET_ was calculated *via* BET (Brunauer–Emmet and Teller) equation.^42^

### 2.6. Column system experiments using industrial wastewater from Emessa Denim Ready Made Garments Factory

The prepared AC and its derivative AC/PA composite were tested on a column system using real industrial wastewater that was collected from Emessa Denim for Ready Made Garments Factory, Industrial Zone, Beni-Suef Governorate, Egypt. The most challenging contaminants that were traced in such industrial wastewater were BOD and COD, which reflect the dye concentration (Table 1). These contaminants were correlated with the bleaching process of jeans pants. However, the other traced contaminants were approximately present within acceptable limits (*i.e.*, fragile in comparison with the dye concentration). So, this study focused only on the essential problem in Factory wastewater, which is industrial dye waste (IDW). In other words, the removal efficiency of both AC and AC/PA composite was evaluated and compared based on the IDW concentration in the effluent water only. The concentration of IDW before and after treatment process was analyzed using a UV-spectrophotometer (DR 6000, HACH LANGE, USA) in the visible region at 671 nm.^43^ In this consequence, the impacts of bed thickness, applied flow rate, and applied pH were investigated for both AC and AC/PA composite.

**Table 1:** Physical and chemical analyses of industrial wastewater from Emessa Denim for Ready Made Garments Factory

Fe (mg l^−1^)	0.10
Pb (mg l^−1^)	0.15
Cd (mg l^−1^)	0.20
Zn (mg l^−1^)	0.25
Cr (mg l^−1^)	0.38
NH_3_ (mg l^−1^)	1–2
BOD (mg l^−1^)	850
COD (mg l^−1^)	1300
TSS (mg l^−1^)	200
TDS (mg l^−1^)	700
pH	7.50
*T* (°C)	25°

#### 2.6.1. Continuous packed bed-adsorption column

There are important parameters needed to check the feasibility of using the prepared materials in industrial applications. The BTC (breakthrough concentration) concept requires analyzing the operating characteristics of a packed bed. The specific shape of the concentration–time profile of BTC, together with the time axis, depends on inlet concentrations, flow rates, and remaining parameters like bed height and diameter of column.^43^ Therefore, BTC analysis is important for the successful design of the fixed-bed adsorption column. Column studies were conducted in a glass column. A known quantity of the addressed materials (AC & AC/PA composite, about 2 mm in size) was fed separately to the column and supported using two layers of cotton wool or sponge, as well as one layer of sand to make the surface more even before the addition of AC and the AC/PA composite. Then, a solution with a known concentration of the industrial dye waste (IDW) was passed through a cylindrical glass tube using a peristaltic pump (Model: PP-20-EX – Miclins, India) in a down-flow mode, as shown in the given schematic diagram (Fig. 2c). Generally, the column was separately filled with calculated quantities (25, 50 & 75 g) of AC and AC/PA composite to achieve a specific bed height (2.5, 5, and 7.5 cm), whereas the other factors were preserved at constant values. In industrial practice, the operating bed height is usually in the range of 0.35–0.50 of the total column height to avoid pressure drop in the column and to maintain the steady state of the applied flow rate. The applied flow rate of the influent solution was controlled using a variable-speed peristaltic pump. Hence, the initial influent flow rate was 5–12 and 20 ml min^−1^ to achieve low, medium, and high flow rates, respectively, keeping the other parameter fixed. The experimental work was carried out at different applied pH (3.0, 7.0 & 9.0) at ambient temperature, while keeping the other experimental factors constant. In each investigated experimental parameter, the effluent samples were collected at specific intervals and analyzed for the IDW concentration using a single beam spectrophotometer (DR 6000, HACH, USA) in the visible region at 671 nm.^43^ Moreover, the zeta potential was measured at room temperature using the Delsa nano C (Beckman Coulter, Switzerland) within the pH range of 2–11 to detect the surface charge of AC and the AC/PA composite. The kinetics models of the adsorption process in a fixed-bed column were also performed using both Thomas and Yoon approximations.

#### 2.6.2. Analysis of column adsorption process

Generally, a plot of *C* effluent (*C*_*t*_) of the applied IDW or *C* effluent/*C* influent (*C*_0_) *vs.* treated volume (*V*) and service time (*t*) at specific bed height, flow rate, and applied pH was used to describe BTC. The evaluation of the BTC trend and the time required for a breakpoint is necessary for the proper design of a fixed bed column. The lower value of concentration, *C*_b_, is selected at the breakthrough point, which is a random value.

## 3. Results and discussion

### 3.1. Characterization of SBC, AC and the AC/PA composite

The XRD of the precursor SBC in comparison with the derivatives AC and AC/PA composite is illustrated in Fig. 2. The broad diffraction background in the derivative ACs, which reflects the gained amorphicity after the precursor SBC, was preserved, but with less magnitude and slight shifting in 2*θ* values (from 20–30° instead of 20–25° in SBC).^44^ However, some signs of improved crystallinity were traced in the derivative AC through the emergence of some peaks at 2*θ* ≈ 17.6° & 23.5°. These latter peaks could be ascribed to the γ-phase carbon.^45^ Meanwhile, the strong diffraction peak at 2*θ* ≈ 25.01° & 26.85° in AC that was recorded as proto-peaks in the precursor SBC (2*θ* ≈ 24.5–26.7°)^46^ indicates the crystalline carbon.^45^ The (002) graphite reflection plane with *d*_(002)_ ≈ 3.32 Å in the derivative AC was reflected by the strong peak at 2*θ* ≈ 26.85°. Such (002) reflection plane approximately vanished in the prepared AC/PA composite, signifying a low degree of graphitization. Therefore, the PA incorporation played a critical role in the reduction of the graphitization degree of AC (*i.e.*, crystallinity slightly reduced). This is in accordance with the applied formic acid as a polymer dissolving agent during the polymerization process.^47^ Thus, the inception of PA into active carbon destroyed the lateral dimension (along the *c*-axis) of nanographene zones, while keeping the longitudinal dimension (along the *a*-axis) intact.^48^ This type of structural disordering has also been reported *via* halidation processes.^48^

**Fig. 3:**
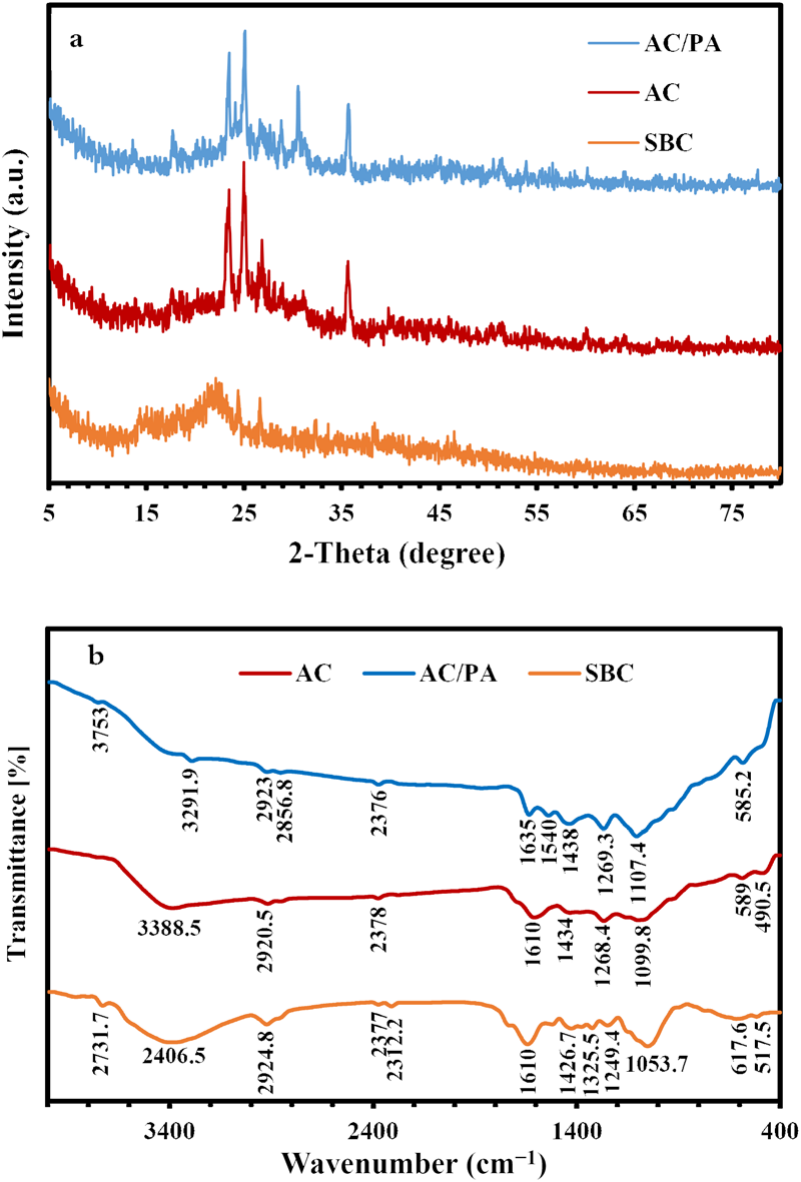
XRD patterns of the prepared AC and AC/PA composite in comparison with the precursor SBC (a). FT-IR spectra of the prepared AC and AC/PA composite in comparison with the precursor SBC (b).

Additionally, the non-carbonaceous phases of the precursor SBC, which were assigned to the residual ash after the activation process in the form of cristobalite, were preserved in the derivatives (AC & AC/PA composite) as very minor peaks at 2*θ* ≈ 51.05° & 60.1° (Fig. 3). Similarly, the strong peaks in the pattern of the derivatives AC and AC/PA composite around 2*θ* ≈ 35.6° were correlated with silicon carbide.

FT-IR spectra of the pristine SBC compared to those of the derivative AC and AC/PA composite are given in Fig. 3. The SBC prevailing functional groups were reported at frequencies of 3406.5, 2924.8, 1634.2, and 1053.7 cm^−1^. The broad absorbance band at 3406.5 cm^−1^ in the SBC spectra might be ascribed to the stretching vibrational mode of either O–H or N–H groups.^49^ In the derivative AC and AC/PA, the peak amplitude of the N–H and/or O–H groups was gradually diminished with progressive shifting to lower frequencies after the thermo-chemical activation and polymerization processes. The less intense band at 2924.8 cm^−1^ of SBC, which was ascribed to the stretching mode of the C–H (alkenes) group of lignin polysaccharides (*i.e.*, hemicellulose and cellulose),^50,51^ also was noticeably decreased in intensity for AC and the AC/PA composite. Such fading indicates the disintegration of the groups expressing the oxygenated hydrocarbons.^44^ The feeble band around 2300 cm^−1^ of the precursor SBC, which was assigned to CO_2_ molecules in the stretching modes,^52,53^ decreased in intensity in the derivatives AC and AC/PA. Similarly, the C=C aromatic bond^54^ and/or the asymmetric stretching modes of the (–COO^−^) group^50^ around 1634.2 cm^−1^ in SBC also were reduced in intensity in both AC and AC/PA, with some shifting to a higher frequency in the latter. Conversely, the intensity in the C=C group stretching mode^55^ that emerged as a shoulder band at 1522.8 cm^−1^ in the precursor SBC spectra was amplified in the derivatives AC and AC/PA, and shifted up to higher frequencies (≈1540 cm^−1^).^52^ This reflects a remarkable improvement in the aromatic characteristics of the prepared AC *via* the aliphatic component's transformation in the pristine SBC.^56^ Moreover, the noticeable increase in intensity of the weak bands at 1434 and 1269 cm^−1^ (bending mode of the CH_2_ group and amide III (γ), respectively) in the derivative composite compared to SBC, indicates the success of activation and polyamide incorporation in the AC structure. The 1053.7 cm^−1^ intensified band in SBC, which was correlated with C–O stretching,^50^ was shifted to higher frequencies in the derivatives AC & AC/PA (≈1099–1107 cm^−1^, respectively), indicating the coupling between the stretching mode of the O–C group within the aromatic P–O–C linkages and hydrogen-bonded P=OOH groups from polyphosphates/and or phosphates.^57,58^ Moreover, the remarkable occurrence of the bands around 589 cm^−1^ in AC confirms the existence of out-of-plane deformation for C–H from benzene and alkene derivatives with various grades of substitution.^59,60^ Similarly, the incorporation of polyamide contributed to an increase in the intensity of the 585.2 cm^−1^ band, signifying the presence of amide VI (α) in the prepared composite.

**Fig. 4:**
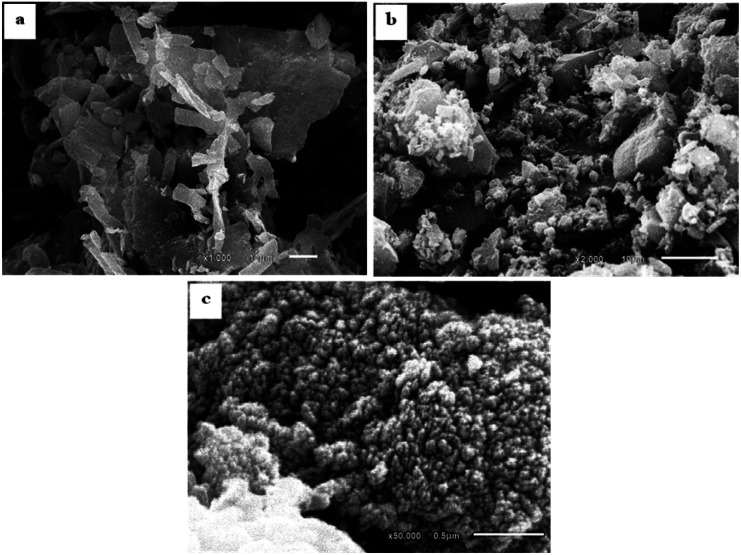
SEM images showing: (a) SBC with nodular crenulations as a reflection of the rough surface; (b and c) AC with a rough surface and agglomerated spheroidal nanoparticles in juxtaposition with well-developed graphite crystals.

**Fig. 5:**
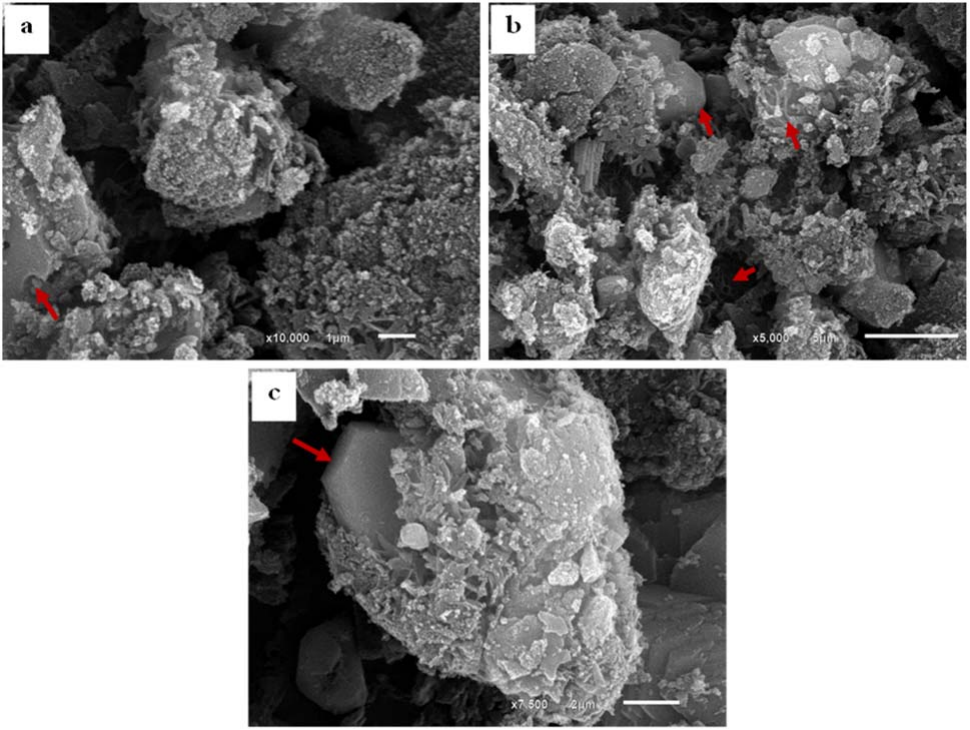
SEM images of the AC/PA composite showing: grooves and cavities produced by formic acid during the polymerization process of AC (a); agglomerated AC with well-developed graphite crystals in association with spherical particles (b and c).

The SEM images of SBC compared to the derivatives AC & AC/PA composites are displayed in Fig. 4. The SBC revealed a rugged surface with spheroidal crenulations (Fig. 4a). The surface heterogeneity was amplified after thermos-chemical activation in the produced AC, as well as the crowding with the spheroidal crenulations (Fig. 4b and c). Such heterogeneity could be accounted for the evaporation of H_3_PO_4_ activating agent during the carbonization process.^56,58^ The presence of some approximately euhedral to sub-euhedral prismatic crystals reflects the attained degree of crystallinity after the carbonization process of the pristine SBC in agreement with XRD results. However, the presence of spherical-like nanoparticles, in juxtaposition with the well-developed crystals, indicates that some traces of the preserved amorphous feature in the AC was maintained. Similarly, the prepared AC/PA composite has a rough/agglomerated surface including spherical-like nanoparticles that generally agglomerate together to generate a porous structure full of grooves and cavities (Fig. 5a–c). Such rough and heterogeneous surfaces with a porous nature were inherited from the precursor AC. Furthermore, the polymerization process not only contributed to agglomeration in the prepared composite, but also maintained some signs of graphitization that reflect the degree of crystallinity of such composite, matching with the XRD data.

Table 2 illustrates the elemental composition of the three materials analyzed by EDS, including the AC/PA composite and each of its components, AC_1:1_ and PA. Carbon (C) and oxygen (O) represent the highest percentages in the three materials with a special preference for the former. Meanwhile, hydrogen (H) and nitrogen (N) have the lowest contribution to the composition of all analyzed materials.

**Table 2:** EDS analysis of the AC/PA composite, and its components of AC_1:1_ and PA

Element (%)	AC_1:1_	PA	AC/PA
C	75.80	74.30	75.53
O	21.20	11.80	20.70
N	0.24	8.50	1.07
H	2.76	5.40	2.70

**Fig. 6:**
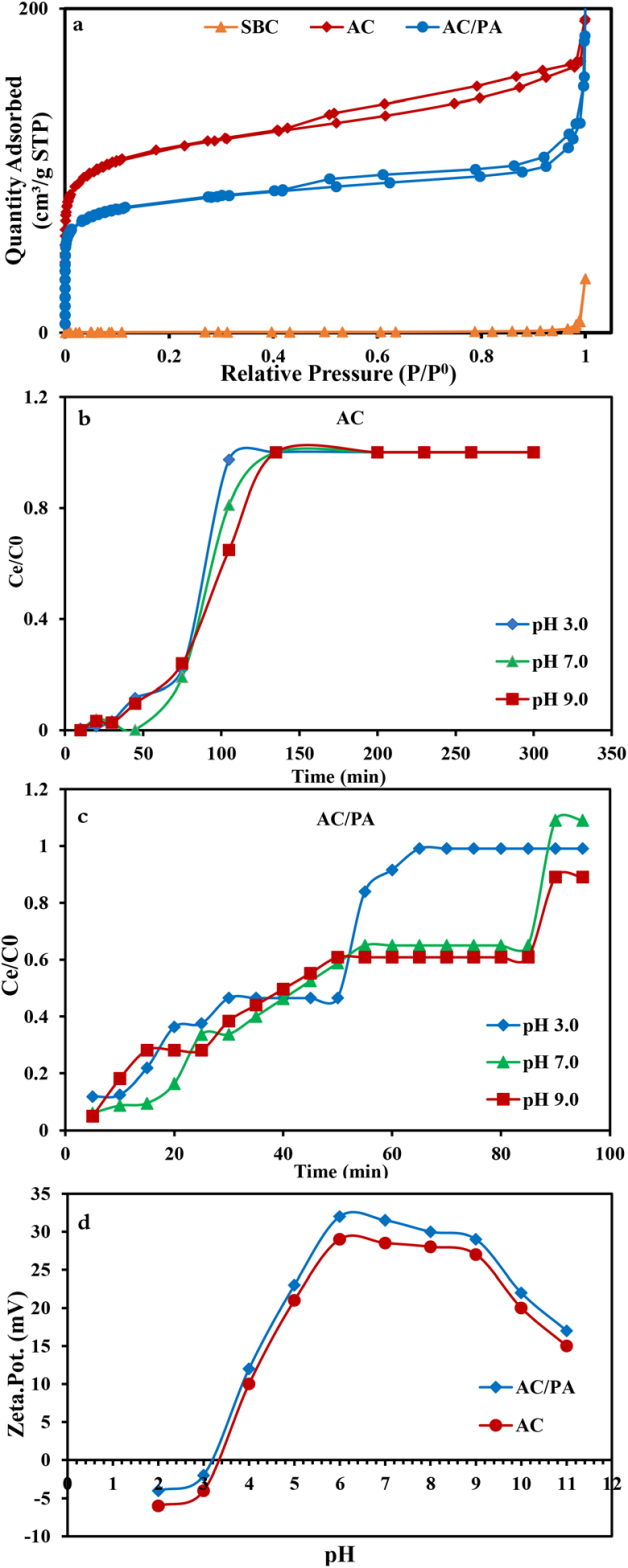
Nitrogen adsorption–desorption isotherms of AC and the AC/PA composite compared to SBC (a). Effect of the applied pH on the breakthrough curves of IDW removal by AC (b), AC/PA composite (c), and the zeta potential of AC and the AC/PA composite (d).

The N_2_ adsorption/desorption isotherms of the parental SBC and the derivatives (AC & AC/PA) are compiled in Fig. 6a. SBC is of type III isotherm consistent with IUPAC classification, whereas the isotherm of hybrid nature (*i.e.*, II and IV) can be designated for the derivatives, AC and the AC/PA composite.^56^ This indicates the macroporous nature of the precursor and macro-porous/mesoporous/micro-porous nature of both derivatives. At low relative pressure (*P*/*P*_0_), the isotherms of the derivatives display the type II nature with remarkable N_2_ adsorption through the available micropores.^56^ Conversely, their isotherms follow the type IV isotherm with H_4_ hysteresis loops, indicating the monolayer–multilayer adsorption.^56^ However, for all of the samples under consideration, the inability to attain the equilibrium status of the N_2_ adsorption confirms the wide scale of the pore diameters, especially for the derivatives. In the same consequence, the geometric parameters of the regarded materials (Table 3) revealed that the carbonization through the thermo-chemical activation process resulted in an observable improvement in the *S*_BET_ and *V*_t_ of the prepared AC compared to those of the precursor SBC (1.5 m^2^ g^−1^ & 0.0107 cm^3^ g^−1^, respectively). This signifies the impact of H_3_PO_4_ in enhancing the porosity of AC through the demolition of the lignin structure/cellulose of SBC.^40^ Additionally, with polyamide incorporation, a slight decline in N_2_ uptake was observed, signaling a slight reduction in the porosity of the prepared AC/PA in comparison with AC. Furthermore, the hysteresis loops were tightened due to the polymerization process and the geometric parameters of the investigated AC/PA (Table 3) witnessed a slim drop in *V*_t_ from 0.26 in AC to 0.02 cm^3^ g^−1^ in the prepared composite. This suggests the role of the polyamide in diminishing the porosity of the prepared AC/PA through the filling of the AC pores.^48^ Concerning *D*_p_, the impact of polyamide incorporation also was reflected in a slight reduction of *D*_p_ to 2.414 nm instead of 2.48 (Table 3). Furthermore, the *S*_BET_ was slightly reduced (from ≈416 to 400 m^2^ g^−1^).

**Table 3:** Textural parameters of the precursor SBC obtained from the nitrogen adsorption isotherms in comparison with the AC and AC/PA derivatives

Sample	Surface area (m^2^ g^−1^)	Total pore volume (cm^3^ g^−1^)	Average pore diameter (nm)
	(*S*_BET)_	*V*_t_	*D*_p_
SBC	1.50	0.0107	28.748
AC_(1:1)_	416.77	0.2584	2.4803
AC/PA	400.32	0.2013	2.4140

### 3.2. Column system experiments using industrial wastewater from Emessa Denim Ready Made Garments Factory using the prepared AC and AC/PA composite

#### 3.2.1. Effect of pH value

The applied pH is one of the most important factors to be considered in adsorption. The changes in pH have significant effects on the adsorption of dye.^61^ The effect of pH on the removal% was evaluated by changing the pH of the IDW solution (490 mg l^−1^) from 3.0 to 9.0 using both AC and AC/PA composite separately at a fixed bed height (5 cm) and flow rate (12 ml min^−1^) (Fig. 6b and c). Concerning AC, the breakthrough points were achieved at 105, 105, and 85 min at pH 9.0, 7.0, and 3.0, respectively. Furthermore, the IDW adsorption revealed an observable increase with increasing applied pH from 3.0 to 9.0 (Fig. 6b). Thus, it can be concluded that in environments with pH below 7.0 (*i.e.*, in the acidic media), the surface of the AC was positively charged, reducing the IDW adsorption.^61^ This matches with the zeta potential results (Fig. 6d) that revealed an isoelectric point of the investigated AC around ≈pH 3.30. Below this value, the AC surface has a negative zeta potential that promoted the protonation process of the surface functional groups, causing a repulsive interaction with IDW ions. However, above pH 3.0 (*i.e.*, at pH 7.0), more adsorption was observed due to the developed negative charge over the adsorbent surface in accordance with the experienced deprotonation process, aligning with the positive zeta potential results. Therefore, the attraction of IDW molecules to the AC was improved, resulting in a higher degree of their adsorption.^62,63^ A marginal decrease in adsorption percentage in the more alkaline region (pH 9.0) may be due to saturation of the available sites on AC, and thus repulsion of IDW in solution by the already adsorbed molecules.^64^ Similar behavior has also been observed in earlier studies. In the case of the AC/PA composite, with the progressive increase in the applied pH, the breakthrough time decreased and became lower than the AC breakthrough time, and the BTCs shifted closer to the origin (Fig. 6c). Breakthrough points were achieved at 85, 85 and 55 min at pH 9.0, 7.0 and 3.0, respectively. At pH 3.0, the surface of AC/PA was also positively charged, so the adsorption capacity of cationic IDW was reduced due to the electrostatic repulsion with the protonated surface of the AC/PA composite. This was supported by the zeta potential data (Fig. 6d) that displayed an isoelectric point of the investigated AC/PA composite at about ≈pH 3.2. Beneath such value, the AC/PA surface is even more negative than that of AC, causing a more severe repulsion with IDW ions. Additionally, higher pH values (around 7.0) resulted in the enhancement of the adsorption capacities. This could be attributed to the electrostatic attraction between the solute and the negatively charged binding sites on the composite surface in accordance with the deprotonation process coupled with a progressive increase in the applied pH, matching with the alignment with the displayed positive zeta potential values. Despite the improvement in the removal% at pH 9.0, the electrostatic interactions have little effect on the sorption process in accordance with the probable degradation and precipitation of the IDW. Therefore, subsequent experiments were performed at pH 7.0.

#### 3.2.2. Effect of flow rate

**Fig. 7:**
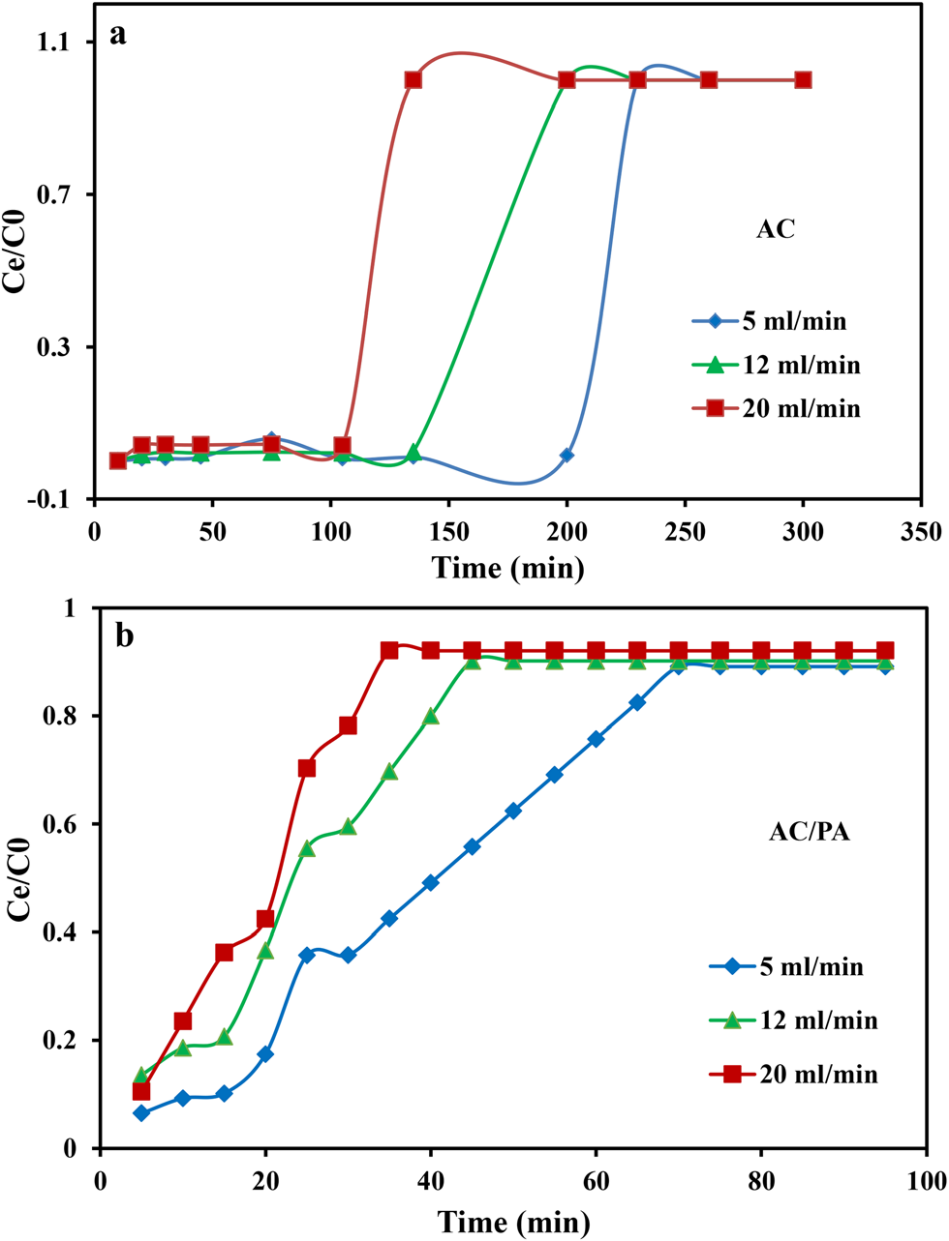
Effect of the flow rate on the breakthrough curves for IDW by AC (a) and the AC/PA nanocomposite (b).

Flow rate is an important parameter for designing the adsorption column, as it determines the contact time of IDW by the investigated adsorbents. In the current study, various flow rates (5, 12, and 20 ml min^−1^) were applied, while keeping the other parameters constant (bed height, IDW concentration, and initial pH at 2.5 cm, 490 mg l^−1^, and 7.0, respectively). The plot of the normalized IDW concentration (*C*_*t*_/*C*_i_) and time (min) at different applied flow rates are given in Fig. 7a and b to highlight the significance of this parameter on the breakthrough capacity. Concerning AC, the progressive increase in the applied flow rate from 5 to 20 ml min^−1^ resulted in a noticeable reduction in the breakthrough time from ≈230 min to ≈135 min, respectively (Fig. 7a). This was attributed to the short residence time at the higher flow rate, leading to limited diffusivity of the IDW ions into the AC active sites. So, a very fast elution of the IDW solution from the column (before attaining an equilibrium state) was observed in accordance with an acceleration in the effluent velocity.^65^ The reduction in the contact time of IDW contributed to the improper execution of the intra-particle diffusion phenomena between IDW molecules and AC. Hence, the adsorption capacity (*q*_total_) decreased with the increase in the applied flow rate.^65,66^ Furthermore, the time required to attain a specific breakthrough concentration was reduced (*i.e.*, rapid occurrence of the BTC) compared to the lower flow rate. BTCs of the AC/PA varied with flow rates. In general, the higher the flow rate, the steeper and the shorter exhaustion time were observed for the BTCs (Fig. 7b). A plausible explanation is that the increase in flow rate from 5 to 20 ml min^−1^ leads to a shorter contact time from 70 to 30 min between the AC/PA particles and the adsorbate ions, and consequently results in an approximately fast arrival of the related BTCs compared with the AC contact time, especially at a higher flow rate (30 min). As the inlet flow rate increases, both the axial dispersion and overall mass transfer coefficient increase,^65^ resulting in an increase in the adsorption rate and faster saturation of the fixed bed. The shorter contact time of AC/PA than AC may be attributed to the higher rate of mass transfer and few active sites available on the surface of AC/PA.

#### 3.2.3. Effect of bed height

**Fig. 8:**
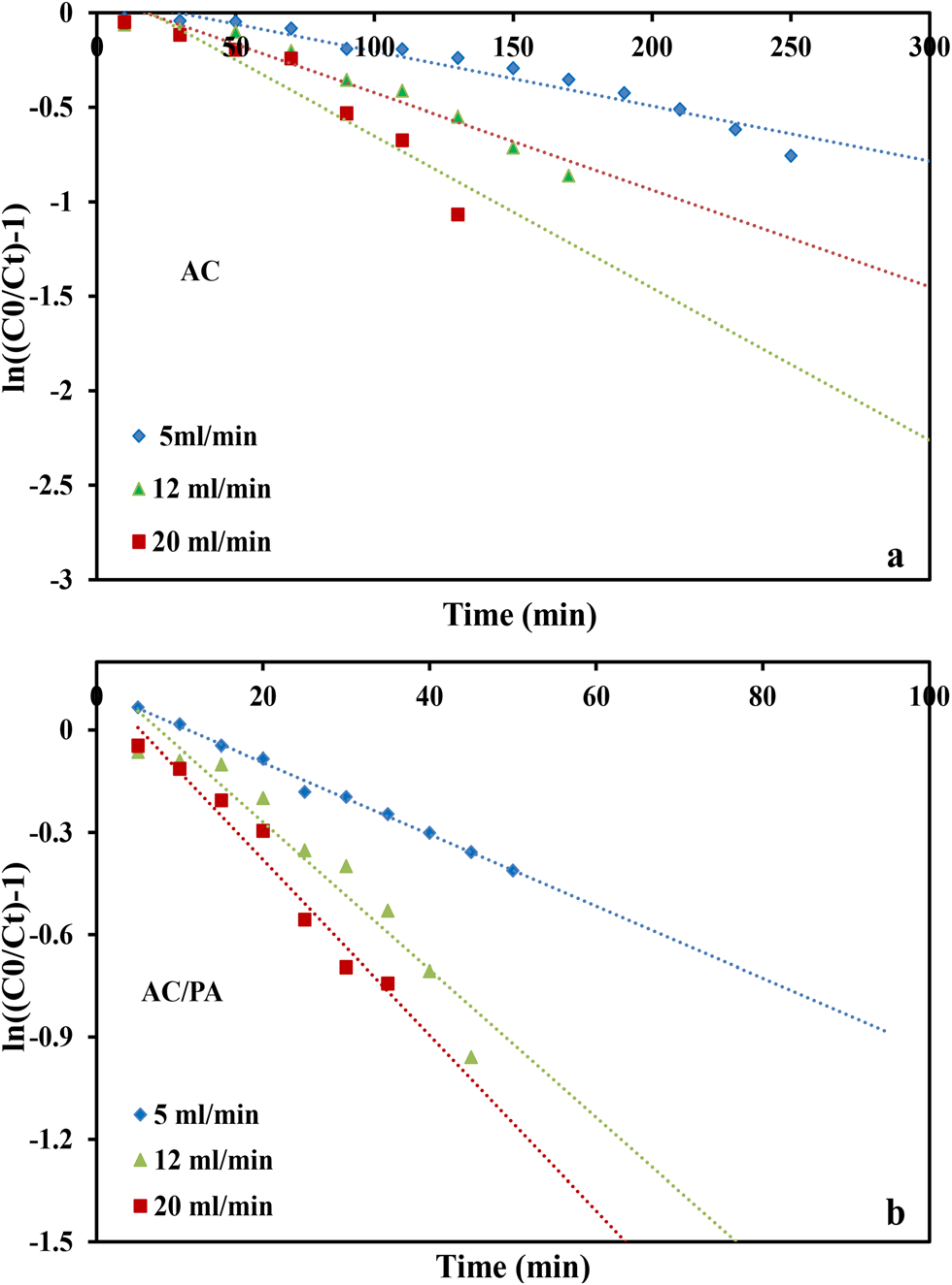
Effect of the bed height on the breakthrough curves for the IDW dye by AC (a) & the AC/PA nanocomposite (b).

The influence of bed heights (2.5, 5 & 7.5 cm) on the adsorption of industrial dye waste (IDW) onto commercial AC was investigated at a constant flow rate of 12 ml min^−1^, IDW concentration of 490 mg l^−1^, and pH around 7.0. Breakthrough curves at different bed heights are shown in Fig. 8a. The increase in the AC bed height from 2.5 to 7.5 cm is accompanied by a noticeable increase in the breakthrough time (*t*_b_) and saturation time (*t*_s_) from 105 to 200 min, respectively. The shape of BTC observed for the bed height of 2.5 cm was steeper than that of 5 and 7.5 cm. This was attributed to the shorter mass transfer zone developed in a column, whereas the steepness of the S curve decreased as the AC bed height increased (Fig. 8a) due to the fact that saturation of the longer bed height takes more time. As the bed height decreased, the load of adsorbent in the column was less, so there was less capacity for the bed to adsorb dye from the solution. However, the overall rate of adsorption was very fast (*i.e.*, Lewis batch-like system behavior was achieved). Also, the dye adsorption capacity increased due to the increase in the accessible binding sites and residence time.^67^ Once the adsorbent was close to saturation state, there was a rise in the concentration of IDW in an effluent stream as time progressed. As the quantity of loaded adsorbent in a column was high, the contact time between the adsorbate–adsorbent was increased, resulting in enhanced “sweep efficiency”. In the case of the AC/PA composite (Fig. 8b), the breakthrough time (25–65 min) was increased by increasing the bed height. A close examination revealed that BTCs shifted towards the origin at smaller bed heights. The observed trend in BTCs can be attributed to the early saturation of the fixed-bed due to the presence of less sorption sites and acceleration of the adsorption rate at smaller bed heights than in the case of AC at the same bed heights.^68^ Furthermore, with an increase in the AC/PA bed height, the adsorption capacity also increased as the adsorbent height was raised from 2.5 to 7.5 cm. This increase may be attributed to the presence of more binding sites, implying a greater diffusion of adsorbate around the solid particles.^69^ However, the increase of the AC/PA bed height is less effective in providing more binding sites and sufficient residence time for the adsorption process in comparison with AC. Therefore, there is less IDW adsorbed on the AC/PA composite in the fixed bed.

### 3.3. Adsorption modeling for fixed bed column studies

#### 3.3.1. Thomas model

**Fig. 9:**
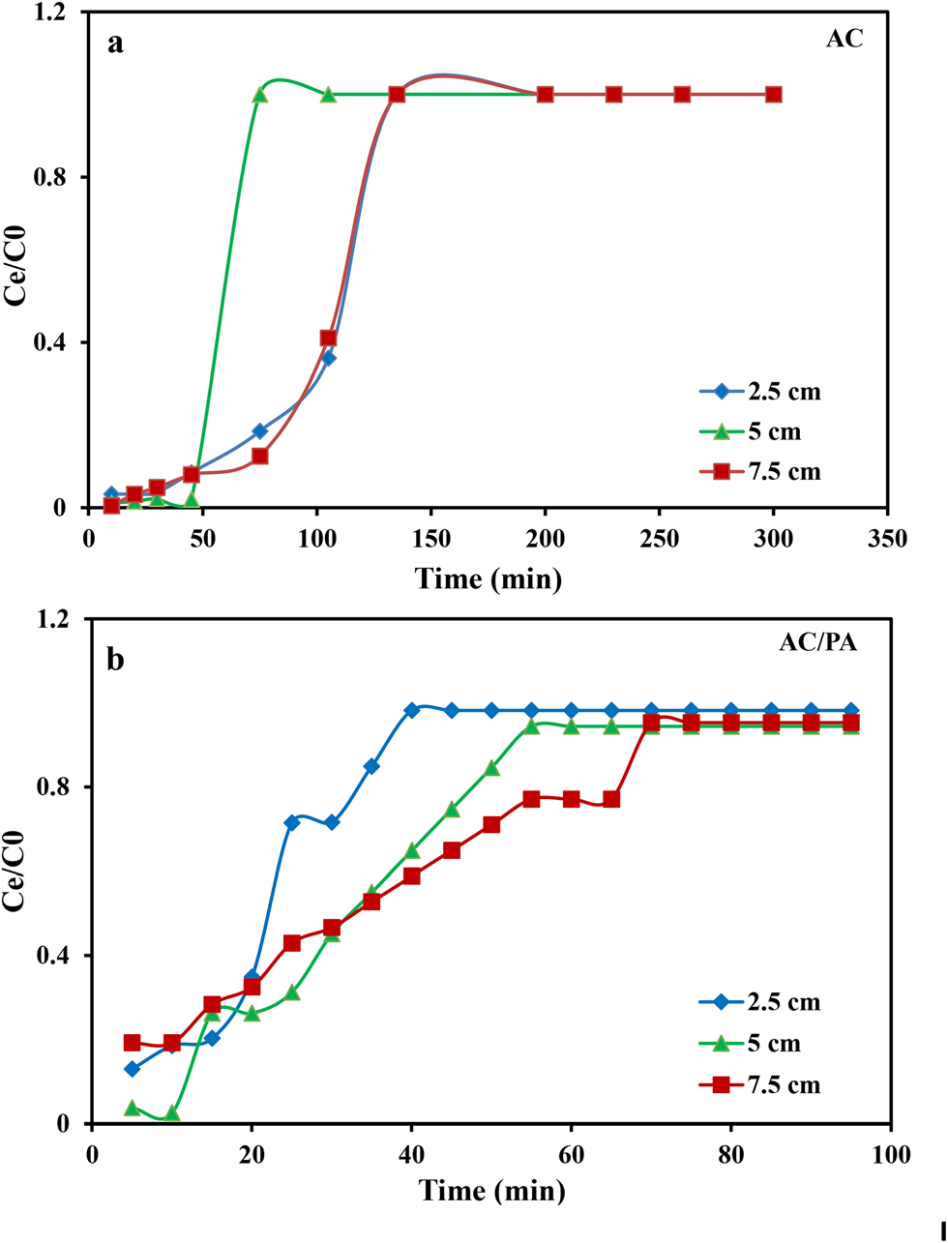
Plots of the Thomas model for AC (a) and the AC/PA nanocomposite (b).

The Thomas model was used to describe the breakthrough of a fixed-bed column and the influencing adsorption parameters of the column system for both AC and the AC/PA composite. This model was expressed through the second-order law of kinetic reaction without the presence of an axial dispersion even when the bed depth was at the minimum and the breakthrough occurred immediately after the flow started.^70^ The Thomas model is expressed in a linearized form as given in the following equation:
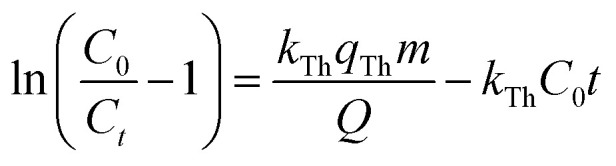
where *k*_Th_ (ml min^−1^ mg^−1^) is the Thomas model constant, *q*_Th_ (mg g^−1^) is the predicted adsorption capacity of IDW by the addressed adsorbents that was calculated *via* the Thomas model, and *m* is the utilized mass of AC and AC/PA adsorbents (g) in the column system, separately. *Q* is the influent flow rate (ml min^−1^), *C*_0_ and *C*_*t*_ are the IDW concentration (mg l^−1^) of the influent and effluent, respectively, and *t* is the time in (min). The values of *k*_Th_ and *q*_Th_ (Table 4) were obtained from the plot of ln(*C*_0_/*C*_*t*_ − 1) *versus t* (min) at various flow rates (5, 12, and 20 ml min^−1^), while keeping the other experimental parameters constant (bed height, IDW concentration and initial pH at 2.5 cm, 490 mg l^−1^ and 7.0, respectively), as shown in Fig. 9a and b. Based on the results of the linear plot of ln(*C*_0_/*C*_*t*_ − 1) *versus t* (min) at the different applied flow rates (Fig. 9a), the high correlation (*R*^2^ > 0.94) of the experimental data revealed that the adsorption process of IDW onto AC and AC/PA could be well-interpreted by the Thomas model (Table 4). Furthermore, the *k*_Th_ values revealed contradictory behavior for both adsorbents. The *k*_Th_ values for the AC/PA composite revealed a progressive increase upon increasing the applied flow rate from 5 to 20 ml min^−1^. Conversely, the *k*_Th_ values were decreased with increasing applied flow rate in the case of AC. However, the *k*_Th_ values of the AC/PA composite are still higher than those of their counterparts of AC at the same applied flow rates. Furthermore, the calculated adsorption capacities (*q*_Th_) revealed reversible trends at an enlarged flow rate for both adsorbents. These results might be attributed to the enhancement of mass transfer driving forces with the decrease in inflow rate in both cases.^67^ On the contrary, the interaction time between IDW and AC & AC/PA gradually decreased with the increase of inflow rate, eventually resulting in the decrease of the sorption amount. However, the recorded *q*_Th_ values using AC are still higher than those of AC/PA at the same applied flow rate. Moreover, the small relative error between the calculated and experimental (average value for the 3 applied flow rates) uptake capacities for both adsorbents suggests the suitability of the Thomas model in predicting the dynamic adsorption process of IDW onto AC and the AC/PA composite (Table 4). These results fully proved the chemisorption process of IDW by both AC and AC/PA adsorbents.

**Table 4:** Thomas and Yoon-Nelson model parameters for AC & the AC/PA composite

	Flow rate (ml min^−1^)	*q*_exp_	Model					
			Thomson			Yoon-Nelson		
			*k*_Th_	*q*_Th_	*R* ^2^	*k*_YN_	*τ*	*R* ^2^
AC	5	439.20 (mg l^−1^)	3.1954 × 10^−6^	450.7	0.976	0.005	92.87	0.876
	12		2.1451 × 10^−6^	401.2	0.976	0.008	31.84	0.729
	20		1.3293 × 10^−6^	366.7	0.948	0.016	21.04	0.921
AC/PA	5	117.30 (mg l^−1^)	1.14548 × 10^−5^	154.9	0.997	0.136	10.92	0.771
	12		1.9314 × 10^−5^	105.0	0.961	0.179	5.61	0.845
	20		2.8633 × 10^−5^	99.68	0.981	0.208	4.01	0.890

#### 3.3.2. Yoon and Nelson model

**Fig. 10:**
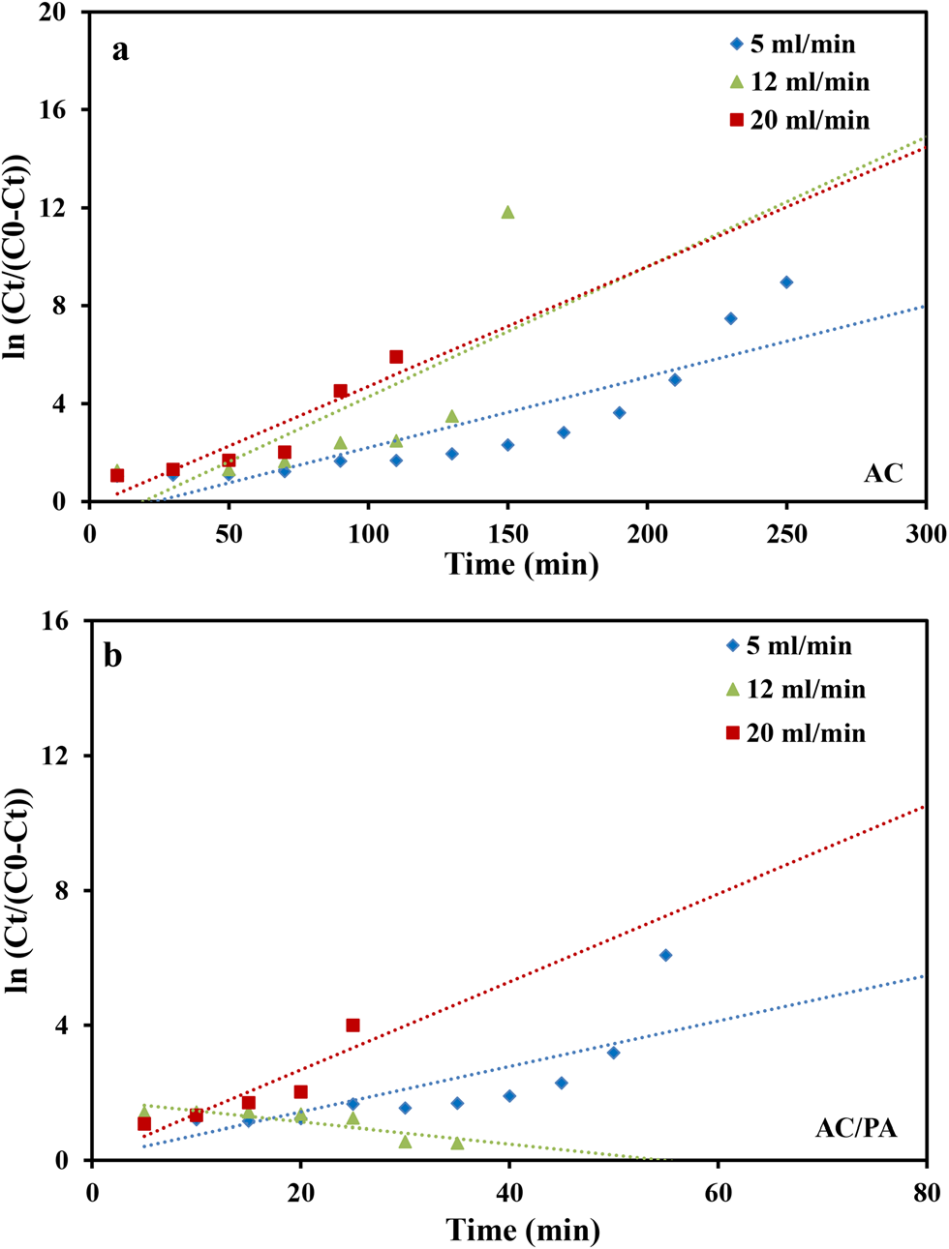
Plots of the Yoon-Nelson model for AC (a) and the AC/PA nanocomposite (b).

The Yoon & Nelson model was developed in 1984 (ref. 71) based on the assumption that the rate of the decrease in the probability of the adsorption for each adsorbate molecule is proportional to the probabilities of the adsorbate adsorption and the adsorbate breakthrough on the adsorbent.^72^ The linear form of the Yoon-Nelson model is expressed in the following equation:
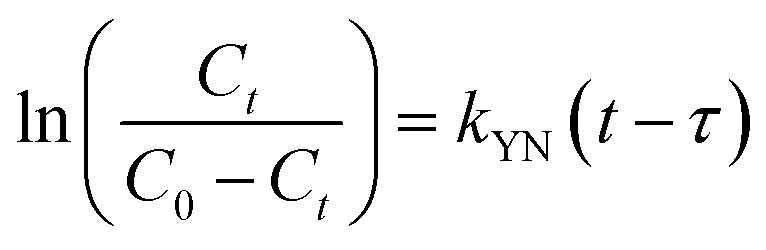
where *k*_YN_ (min^−1^) is the Yoon and Nelson rate constant and *τ* (min) is the half-breakthrough time. The *k*_YN_ and *τ* terms were calculated using the intercept and linear drawing slope of ln(*C*_*t*_/*C*_0_ − *C*_*t*_) *versus* (*t*). From the linear plot of ln(*C*_*t*_/C_0_ − *C*_*t*_) *versus* (*t*) at the different applied flow rates (Fig. 10a and b), the recorded determination coefficients *R*^2^ (from 0.73 to 0.92 & 0.77 to 0.89 for AC and AC/PA, respectively) indicate that the adsorption process of IDW onto AC and AC/PA could not be interpreted well using the Yoon and Nelson model compared to the Thomson one (Table 4).

## 4. Conclusions

Grounding upon the above-mentioned discussions, the following deduction can be formulated:

• SBC waste that annually piles up after the harvesting of beet crop was converted into sustainable AC with high market value by thermo-chemical activating procedure using a fixed weight ratio of the H_3_PO_4_ acid : SBC (1 : 1) and 550 °C for 2 h.

• The polymerization process of the fabricated AC (90%) with polyamide (PA, 10%), as synthetic textile waste, using an adequate ratio of the specified dissolving agent and the employed polymer (formic acid/PA = 82%/18% w/w ratio), resulted in the production of the AC/PA nanocomposite.

• The remarkable surface chemistry and geometric parameters of both AC and AC/PA nanocomposite (*e.g.*, *S*_BET_ = 416.77 and 400.32 mg g^−1^, respectively) made these materials a suitable and effective choice for IDW remediation from the industrial wastewater effluent of the Emessa Denim Ready Made Garments Factory.

• The sorption of IDW by regarded adsorbents was a pH-flow rate and bed thickness driven procedure; the maximum removal percentage was accomplished at pH 7.0 & 9.0, matching with the zeta potential findings. Moreover, the equilibrium was reached at 60 and 15 min, respectively.

• The reduction of the retention time contributed to the improper execution of the intra-particle diffusion phenomena between IDW molecules and binding site on the surface of the regarded adsorbents, reducing the required time to attain a specific breakthrough, especially at high flow rates and low bed thicknesses as a consequence of the limited diffusivity of the IDW ions into the binding sites of the adsorbents.

• The shorter breakthrough time of AC/PA than AC may be attributed to the higher rate of mass transfer and the reduction in the available binding sites on the surface of the composite as a result of the polymerization process.

• Both experimental and calculated adsorption capacities (*q*_Th_) revealed reversible trends at enlarged flow rates for both AC and AC/PA with some superiority of the former over the latter. These results might be attributed to the enhancement of mass transfer driving forces with the decrease of inflow rate in both cases.

• The small relative error between the calculated and experimental uptake capacities for both adsorbents suggests the suitability of the Thomas model in predicting the dynamic adsorption process of IDW onto AC and the AC/PA composite.

• IDW adsorption by both AC and AC/PA adsorbents was a chemisorption process.

• Finally, AC and AC/PA can be classified as excellent/eco-friendly adsorbents for dyes from industrial wastewater with some advantages for the former.

## Conflicts of interest

The authors declare no conflict of interest.

## Supplementary Material
